# Value of Thrombus Imaging Characteristics as a Guide for First‐Line Endovascular Thrombectomy Device in Patients With Acute Ischemic Stroke

**DOI:** 10.1161/SVIN.122.000450

**Published:** 2022-09-09

**Authors:** Nikki Boodt, Agnetha A. E. Bruggeman, Manon Kappelhof, Sanne J. den Hartog, Nerea Arrarte Terreros, Jasper M. Martens, Reinoud P. H. Bokkers, Pieter‐Jan van Doormaal, Charles B. L. M. Majoie, Wim H. van Zwam, Henk A. Marquering, Diederik W. J. Dippel, Aad van der Lugt, Hester F. Lingsma, the MR CLEAN Registry Investigators

**Affiliations:** ^1^ Department of Radiology and Nuclear Medicine Erasmus MC, University Medical Center Rotterdam Rotterdam The Netherlands; ^2^ Department of Neurology Erasmus MC, University Medical Center Rotterdam Rotterdam The Netherlands; ^3^ Department of Public Health Erasmus MC, University Medical Center Rotterdam Rotterdam The Netherlands; ^4^ Department of Radiology and Nuclear Medicine Amsterdam UMC, University of Amsterdam Amsterdam The Netherlands; ^5^ Department of Biomedical Engineering and Physics Amsterdam UMC, University of Amsterdam Amsterdam The Netherlands; ^6^ Department of Radiology and Nuclear Medicine Rijnstate Hospital Arnhem The Netherlands; ^7^ Medical Imaging Center, Department of Radiology Groningen University of Groningen University Medical Center Groningen Groningen The Netherlands; ^8^ Department of Radiology and Nuclear Medicine Cardiovascular Research Institute Maastricht (CARIM) Maastricht University Medical Center Maastricht The Netherlands

**Keywords:** acute stroke, aspiration, stent retriever, thrombectomy, thrombus

## Abstract

**Background:**

It has been suggested that selection of a first‐line endovascular thrombectomy device, that is, contact aspiration (CA) or stent retriever (SR) thrombectomy, could be based on thrombus type. Thrombus composition and mechanical behavior can partially be predicted with thrombus computed tomography (CT) characteristics. We aimed to assess the influence of thrombus CT characteristics on the association between first‐line device and outcomes of endovascular thrombectomy.

**Methods:**

For patients enrolled in the MR CLEAN (Multicenter Randomized Clinical Trial of Endovascular Treatment for Acute Ischemic Stroke in The Netherlands) Registry between March 2014 and November 2017, we assessed thrombus density, thrombus length, and presence of hyperdense artery sign on thin‐slice (≤2.5 mm) admission CT. We used regression models to estimate the relationship between first‐line endovascular thrombectomy device (CA versus stent retriever) and first‐pass reperfusion (FPR, expanded Thrombolysis in Cerebral Infarction score 2C‐3 after first attempt), final reperfusion, procedure duration, 24‐hour National Institutes of Health Stroke Scale, and 90‐day modified Rankin scale score and tested for interaction of thrombus characteristics with first‐line device by adding interaction terms.

**Results:**

Of 703 included patients, 520 (74%) received first‐line stent retriever and 183 (26%) first‐line CA. Overall, the first‐line device was not associated with FPR (adjusted odds ratio [aOR], 1.32 [95% CI, 0.88–1.98]). In patients with thrombus density below the median (<50 Hounsfield units), FPR was more often achieved with CA than with a stent retriever (34% versus 24%, aOR, 1.95 [95% CI, 1.09–3.50]), whereas in patients with thrombus density above the median (≥50 Hounsfield units), first‐line device was not associated with FPR (aOR, 0.90 [95% CI, 0.50–1.62]). The interaction between thrombus density as a continuous variable and first‐line device on outcome was not significant (*P*=0.38). There was also no interaction between first‐line device and the other thrombus characteristics for FPR or the other outcomes.

**Conclusion:**

Our study does not provide evidence that the association between first‐line thrombectomy device and endovascular thrombectomy outcomes depends on thrombus CT characteristics. Based on our results, there are no arguments for thrombectomy device selection based on thrombus CT characteristics. A possible better performance of CA in low‐density, fibrin‐rich clots needs further study.

## Graphical abstract

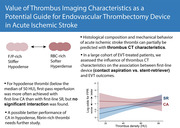

**Nonstandard Abbreviations and Acronyms**

CA: contact aspiration
CTA: computed tomography angiography
DSA: digital subtraction angiography
eTICI: Expanded Thrombolysis in Cerebral Infarction
EVT: endovascular thrombectomy
FPR: first‐pass reperfusion
HAS: hyperdense artery sign
HU: Hounsfield units
MR CLEAN: Multicenter Randomized Clinical Trial of Endovascular Treatment for Acute Ischemic Stroke in The Netherlands
NCCT: noncontrast computed tomography
SR: stent retriever

**Clinical Perspective**

- In a large cohort of patients who underwent endovascular thrombectomy in clinical practice, we found no significant interaction between first‐line thrombectomy device and thrombus computed tomography characteristics on outcomes of endovascular thrombectomy.
- A possible better performance of contact aspiration in low‐density, fibrin‐rich clots needs further study.

Endovascular thrombectomy (EVT) for patients with acute ischemic stroke attributed to large vessel occlusion of the anterior circulation is highly effective.^1^ In the first 5 positive trials, EVT was predominantly performed with stent retrievers (SRs). Since then, clinical trials have shown that EVT with contact aspiration (CA) results in similar angiographic and clinical outcomes as SR thrombectomy.^2^, ^3^

After EVT became the standard of care in 2015, research has increasingly focused on improving the rates of fast and complete reperfusion. Thrombus subtype may play a key role in achieving benchmark reperfusion outcomes. In vitro studies have shown that fibrin‐rich thrombi are stiffer than red blood cell–rich thrombi,^4^, ^5^, ^6^ leading to more friction between the thrombus and vessel wall^7^ and less integration with the SR.^8^ In line with these findings, clinical studies have shown that with SRs, fibrin‐rich thrombi are associated with more thrombectomy attempts, longer procedures, and lower reperfusion scores than red blood cell–rich thrombi.^9^, ^10^ In contrast to SR thrombectomy, CA only interacts with the proximal interface of the thrombus and does not rely on integration of the device with the thrombus along its length. Therefore, it has been suggested that CA might be a better approach for fibrin‐rich, thrombectomy‐resistant thrombi. Indeed, a recent study found a significant interaction between presence of the hyperdense artery sign (HAS), indicating red blood cell–rich thrombus,^11^, ^12^ and first‐line device: in patients with HAS, better reperfusion was achieved when an SR was used as a first‐line approach, whereas patients without HAS had better outcomes with first‐line CA.^13^

There is no standardized method of assessing HAS presence. Therefore, quantified thrombus imaging characteristics have the potential to provide a more accurate representation of thrombus subtype. Thrombus density and thrombus length on admission computed tomography (CT) are strongly associated with thrombus composition,^14^ but their potential impact on the association between first‐line EVT device and outcomes of EVT is unknown. In recent studies, first‐pass reperfusion (FPR, Expanded Thrombolysis in Cerebral Infarction [eTICI] score 2C‐3 after the first attempt) was associated with better clinical outcomes after EVT and is currently often used as a key metric for the efficacy of thrombectomy devices.^15^, ^16^

In this study, we aim to assess the influence of thrombus CT characteristics on the association of a first‐line device (CA versus SR thrombectomy) with FPR and other EVT outcomes in a large, prospective database of patients with acute ischemic stroke who underwent EVT.

## Methods

We used data from the MR CLEAN (Multicenter Randomized Clinical Trial of Endovascular Treatment for Acute Ischemic Stroke in The Netherlands) registry, which was a nationwide, observational, prospective study of all patients who underwent EVT for acute ischemic stroke in The Netherlands.^17^ All patients undergoing EVT (defined as at least receiving arterial puncture) for acute ischemic stroke in the anterior or posterior circulation in 1 of the 17 centers performing EVT in The Netherlands were registered. The central medical ethics committee of the Erasmus Medical Center Rotterdam, The Netherlands, evaluated the study protocol and granted permission to carry out the study as a registry (MEC‐2014‐235). Because of privacy and data safety regulations, the original data are not available for this study. However, syntax files and output of statistical analyses in STATA are available from the corresponding author on reasonable request.

### Patients

For the purpose of this study, we used data from patients included between March 16, 2014, and November 1, 2017, adhering to the following inclusion criteria: aged ≥18 years, treatment in a MR CLEAN trial center, a proximal intracranial arterial occlusion in the anterior circulation (intracranial carotid artery [ICA, ICA‐terminus] or middle cerebral artery [M1, M2]) as shown on CT angiography (CTA), groin puncture within 6.5 hours after stroke symptom onset, and treated with first‐line SR or CA thrombectomy. To allow for the assessment of quantified thrombus CT characteristics, thin‐slice (≤2.5 mm) noncontrast CT (NCCT) and CTA, acquired within 30 minutes from each other, had to be available.

### Endovascular Thrombectomy

The choice of first‐line thrombectomy modality was left to the discretion of the local interventionists. First‐line SR thrombectomy was defined as the use of any SR as first attempt. First‐line CA was defined as the use of any large‐bore aspiration catheter, without the use of an SR, for the first thrombectomy attempt. Simultaneous aspiration on 1 of the catheters during SR thrombectomy was not recorded separately. Therefore, these patients were included in the first‐line SR group.

### Imaging Characteristics

All patients underwent NCCT and CTA at baseline according to local stroke protocols and digital subtraction angiography (DSA) during EVT. The imaging parameters of interest for the current study were absolute thrombus density, thrombus length, and HAS presence.

An imaging core laboratory consisting of 21 observers (20 interventional neuroradiologists and 1 interventional neurologist, blinded for all clinical data except symptom side) assessed HAS presence, occlusion location, collateral score, Alberta Program Early CT score on baseline NCCT, and CTA.^17^ HAS presence was visually assessed. Reperfusion was assessed by the core laboratory on DSA according to the expanded eTICI score.^18^

NCCT and CTA images were aligned with rigid coregistration using Elastix.^19^ Absolute thrombus density and thrombus length were assessed according to previously described methods.^20^, ^21^ A total of 3 spherical markers with a 1‐mm radius were placed in the proximal, middle, and distal parts of the thrombus on coregistered NCCT and CTA. Thrombus density was defined as the mean density (in Hounsfield units [HU]) of the 3 NCCT markers. In addition, 1 marker was placed at the proximal thrombus border and 1 marker at the distal thrombus border. Thrombus length was defined as the largest extension of the contrast filling defect in the occluded vessel on CTA (in mm). If the proximal or distal borders of the thrombus could not be depicted on CTA, thrombus length was based on the HAS on the coregistered NCCT. Measurements were performed by 7 trained raters (M.K., A.A.E.B., N.B., P.R.K., N.A.T., J.W.H., and J.B.) blinded to all other clinical and imaging information.

### Outcome Measures

The primary outcome was FPR, defined as a single pass of the device without rescue treatment with intra‐arterial thrombolytics, resulting in complete or near‐complete reperfusion of the large vessel occlusion and its downstream territory, eTICI 2C‐3.^15^ Secondary outcomes were final reperfusion score (eTICI on the last angiography run), duration of procedure (groin puncture to end of EVT [time of angioseal] in minutes), National Institutes of Health Stroke Scale at 24 hours, and modified Rankin scale score at 90 days.

### Missing Data

For the regression analyses, missing data were imputed using multiple imputation in STATA based on relevant covariates and outcomes. All missing eTICI scores were imputed. Because reperfusion grade can only be reliably assessed when both antero‐posterior and lateral views on postintervention DSA are available,^22^ reperfusion scores of patients assessed in a single projection (antero‐posterior or lateral only) that were scored as eTICI ≥2A were recoded as missing and imputed.

### Statistical Analysis

We compared baseline, imaging, and treatment characteristics of patients with first‐line SR versus CA using descriptive statistics. The association of first‐line thrombectomy modality (CA versus SR) with the primary and secondary outcomes was assessed with univariable and multivariable logistic and linear regression. Thereafter, the effect of thrombus CT characteristics (thrombus density, thrombus length, and HAS presence) on these relationships was investigated by adding interaction terms to the model (first‐line modality×CT characteristic). Thrombus density and thrombus length were tested as continuous variables. For visualization only, we presented the association of first‐line device with the primary and secondary outcomes for subgroups, defined as above and below the median of thrombus density and thrombus length. Based on previous studies and clinical knowledge, we adjusted all of our multivariable models for age, sex, prestroke modified Rankin scale, baseline National Institutes of Health Stroke Scale, occlusion location, collaterals, intravenous alteplase treatment, time from onset to groin puncture, calendar year, and intervention center. Lastly, we visualized the relationship of thrombus imaging characteristics with the primary outcome (FPR) stratified by first‐line thrombectomy device with adjusted margins plots. All statistical analyses were performed with STATA/SE 17.0 (StataCorp, College Station, TX).

## Results

During the inclusion period, 3637 consecutive patients were registered. After excluding patients based on the prespecified criteria (n=481), because no EVT was performed as a result of spontaneous reperfusion on DSA before EVT or because access to the intracranial vasculature was not achieved (n=463), performed procedure was not EVT (n=8), first‐line device was not SR thrombectomy or CA (n=104), first‐line device was missing (n=137), or because suitable thin‐slice imaging was not available (n=1741), a total of 703 patients qualified for the study (Supporting Information Figure S1). In 62 of 703 patients (9%), we could not classify FPR because of missing number of attempts (n=46), because the eTICI score was missing (n=5), or because the eTICI score was assessed on 1 view of the postintervention DSA and therefore recoded as missing (n=11).

Of the included 703 patients, the median age was 71 years (interquartile range [IQR], 61–80 years), 362 (52%) were men, and baseline National Institutes of Health Stroke Scale was 16 (IQR, 11–20) (Table 1). HAS was reported in 386 (55%) patients, median thrombus density was 50 HU (IQR, 44–57 HU), and median thrombus length was 19 mm (IQR, 12–30 mm) (Supporting Information Figure S2). A total of 520 patients (74%) were treated with first‐line SR and 183 patients (26%) with first‐line CA. Patients treated with first‐line CA had slightly lower median thrombus density (49 versus 51 HU). Patients with first‐line CA more often received intravenous alteplase before EVT (79% versus 70%) and more often underwent general anesthesia during the procedure (48% versus 28%). Use of first‐line CA increased during the inclusion period and varied per intervention center (Supporting Information Figure S3).

**Table 1:** Baseline Characteristics of Patients Treated With First‐Line Stent Retriever Versus First‐Line Contact Aspiration

	Missing	First‐line stent retriever (n=520)	First‐line contact aspiration (n=183)	Total (N=703)
Age, y	0/703	71 (61–80)	71 (62–78)	71 (61–80)
Men	0/703	270 (52)	92 (50)	362 (52)
Baseline NIHSS	12/703	15 (11–19)	17 (12–20)	16 (11–20)
Prestroke mRS	13/703			
0		357 (70)	119 (67)	476 (69)
1		62 (12)	18 (10)	80 (12)
2		37 (7)	17 (10)	54 (8)
3 or more		56 (11)	24 (13)	80 (12)
Imaging characteristics				
ASPECTS	1/703	9 (7–10)	9 (7–10)	9 (7–10)
Occlusion location	7/703			
ICA		13 (3)	7 (4)	20 (3)
ICA‐terminus		101 (20)	37 (20)	138 (20)
M1		319 (62)	120 (66)	439 (63)
M2		81 (16)	18 (10)	99 (14)
Collaterals	11/703			
Grade 0		34 (7)	15 (8)	49 (7)
Grade 1		179 (35)	78 (43)	257 (37)
Grade 2		215 (42)	67 (37)	282 (41)
Grade 3		84 (16)	20 (11)	104 (15)
Hyperdense artery sign	6/703	275 (54)	111 (61)	386 (55)
Thrombus density, HU	0/703	51 (44–58)	49 (43–56)	50 (44–57)
Thrombus length, mm	0/703	18 (12–28)	22 (12–34)	19 (12–30)
Workflow and treatment				
Intravenous alteplase	1/703	362 (70)	144 (79)	506 (72)
Time from stroke onset to groin puncture, min	2/703	183 (139–244)	185 (130–250)	185 (138–245)
General anesthesia	29/703	138 (28)	86 (48)	224 (33)
Use of balloon guide catheter*	165/703	305 (73)	31 (27)	336 (63)
Switch to other modality^†^	46/703	92 (18)	41 (22)	133 (19)
Total number of thrombectomy attempts	46/703			
1		191 (39)	82 (49)	273 (42)
2		134 (28)	29 (18)	163 (25)
3		69 (14)	29 (18)	98 (15)
>3		97 (20)	26 (16)	123 (19)
Embolization in new territory	55/703	23 (5)	5 (3)	28 (4)
Outcomes				
FPR	62/703	124 (26)	48 (30)	172
Final eTICI	46/703	4 (2–5)	4 (3–5)	4 (2–5)
Duration of procedure, min	54/703	61 (41–89)	47 (31–68)	57 (40–84)
NIHSS at 24 h	58/703	8 (3–16)	11 (4–16)	9 (3–16)
mRS at 90 d	48/703	3 (2–6)	3 (2–6)	3 (2–6)

Continuous data are presented as median (interquartile range) and categorical data are presented as number (percentage). ASPECTS indicates Alberta Stroke Programme Early CT Score, eTICI, Expanded Thrombolysis in Cerebral Infarction, FPR, first‐pass reperfusion, HU, Hounsfield units, ICA, internal carotid artery, M1/M2, middle cerebral artery, mRS, modified Rankin scale, and NIHSS, National Institutes of Health Stroke Scale.

^*^ Use of balloon guide catheter is often part of the stent retriever thrombectomy procedure.

^†^ Switch from stent retriever to contact aspiration in the first‐line stent retriever group or switch from contact aspiration to stent retriever in first‐line contact aspiration group.

**Table 2:** Primary and Secondary Outcomes for Patients Treated With Contact Aspiration Versus Stent Retriever (Reference) for Subgroups of Thrombus Density and Thrombus Length

Outcome	Effect estimate	Thrombus density <50 HU (n=335)	Thrombus density ≥50 HU (n=368)	*P* for interaction	Thrombus length <19 mm (n=359)	Thrombus length ≥19 mm (n=344)	*P* for interaction
FPR	Odds ratio	1.95 (1.09 to 3.50)	0.90 (0.50 to 1.62)	0.38	1.42 (0.81 to 2.49)	1.17 (0.64 to 2.13)	0.80
Final eTICI	Common odds ratio	1.20 (0.75 to 1.92)	0.89 (0.54 to 1.46)	0.56	1.21 (0.71 to 2.07)	0.91 (0.59 to 1.40)	0.53
Duration of procedure, min	β	−16.2 (−24.4 to −8.0)	−11.4 (−20.1 to −2.7)	0.78	−14.1 (−23.0 to −5.2)	−13.2 (−21.5 to −4.9)	0.98
NIHSS at 24 h	β	1.90 (0.06 to 3.74)	−1.38 (−3.17 to 0.41)	0.35	0.39 (−1.44 to 2.22)	0.01 (−1.70 to 1.72)	0.88
mRS at 90 d	Common odds ratio	0.93 (0.50 to 1.74)	1.44 (0.72 to 2.86)	0.15	1.15 (0.57 to 2.33)	1.16 (0.62 to 2.15)	0.31

Outcomes are shown for subgroups of thrombus density and length split on the median value, whereas interactions were assessed with thrombus density and length as continuous variables. Analyses were adjusted for age, sex, prestroke mRS, baseline NIHSS, occlusion location, collaterals, intravenous alteplase treatment, time from onset to groin, calendar year, and intervention center. Duration of procedure indicates time from groin puncture to eTICI ≥2B or higher or last angiography run in case eTICI ≥2B was not achieved, eTICI, Expanded Thrombolysis in Cerebral Infarction; FPR, first‐pass reperfusion, HU, Hounsfield units, mRS, modified Rankin scale, and NIHSS, National Institutes of Health Stroke Scale.

Overall, FPR was reported in 172 (27%) patients. In the first‐line SR group, FPR was reported in 124 (26%) patients versus 162 (30%) in the first‐line CA group (Table 1). First‐line thrombectomy device was not associated with FPR in the unadjusted and adjusted regression analyses (adjusted odds ratio [aOR] for CA compared with SR, 1.32 [95% CI, 0.88–1.98]) (Supporting Information Table S1). Procedure duration was shorter in the first‐line CA group (aβ, −14 minutes [95% CI, −21 to −8]), whereas first‐line device was not associated with any of the other secondary outcomes.

In the subgroup with thrombus density below the median (<50 HU), FPR was more often achieved with first‐line CA than with first‐line SR thrombectomy (34% versus 24%, aOR, 1.95 [95% CI, 1.09–3.50]), whereas in the subgroup with thrombus density above the median (≥50 HU), first‐line device was not associated with FPR (aOR, 0.90 [95% CI, 0.50–1.62]). However, there was no significant interaction between thrombus density (as a continuous variable) and first‐line device for FPR (*P*=0.38, Figure [A]) or for any of the secondary outcomes (Table 2). There was no interaction between thrombus length and first‐line device for FPR (*P*=0.80, Figure [B]) or any of the secondary outcomes (Table 2) and no interaction of HAS presence for the primary and secondary outcomes (Supporting Information Table S2, Figure S4).

**Figure 1:**
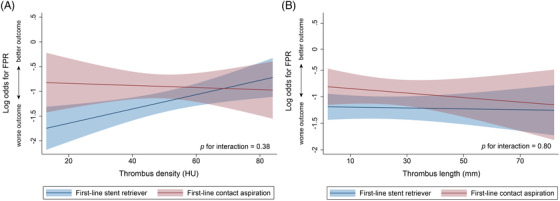
Adjusted margins plots of the relationship between (A) thrombus density and (B) thrombus length and first‐line thrombectomy modality with the primary outcome, first‐pass reperfusion (FPR) are shown.

## Discussion

In this study, we assessed the influence of thrombus CT characteristics on the association of first‐line thrombectomy device and outcomes of EVT in a large registry of current clinical practice. Based on results from previous in vitro and clinical studies, we hypothesized that better outcomes would be achieved with first‐line CA in hypodense, fibrin‐rich thrombi and better outcomes with first‐line SRs in hyperdense, red blood cell–rich thrombi. However, we did not find evidence of an interaction between thrombus CT characteristics and first‐line device on reperfusion outcomes and clinical outcomes of EVT in our data.

In line with our hypothesis, patients with hypodense thrombi, indicating more fibrin‐rich thrombus, had higher rates of FPR with first‐line CA than with first‐line SR. However, we found no interaction between thrombus density and first‐line device on FPR or on the secondary outcomes. It has been shown that interactions are generally hard to study because most studies are underpowered to detect them.^23^ Therefore, our study could have been underpowered to detect an interaction between thrombus density and results of first‐line thrombectomy device. Moreover, thrombus density is a surrogate for thrombus composition. As we are primarily interested in the interaction between first‐line device and thrombus composition, the correlation between density and composition might have been too weak to detect an interaction. Future studies could focus on improving imaging methods for accurate characterization of thrombus composition, such as with dual‐energy CT.

We found no interaction of thrombus length and presence of (visually scored) HAS with first‐line device on FPR, which is in contrast with the results of Mohammaden et al, who reported a significant interaction between HAS and first‐line device for FPR.^13^ A significant limitation of HAS as a potential biomarker for first‐line device is that there is no standardized way of assessing HAS presence, which could lead to significant interobserver variability. This, and the fact that the former study did not use an independent imaging core laboratory, might be an explanation for our contrasting results.

Overall, we found no significant differences in angiographic and clinical outcomes for first‐line CA versus first‐line SR thrombectomy. In line with previous studies, duration of the endovascular procedure was 14 minutes shorter in the first‐line CA group compared with the first‐line SR group.^2^, ^3^, ^24^

Our study has limitations. First, the choice of first‐line thrombectomy device was not randomized. Although we assumed that the interventionalists selected a first‐line device based on personal preference, device selection might have been influenced by admission imaging characteristics, such as HAS. However, there were no large differences between imaging characteristics of patients with first‐line CA versus first‐line SR thrombectomy. Second, information on the additional use of CA for combination therapy was not recorded, therefore, these patients were included in the first‐line SR group. During the inclusion period of 2014 to 2017, CA and SR devices and neurointerventionalist experience with these devices have evolved significantly, potentially leading to better outcomes over time. However, we adjusted our models for calendar year. Lastly, device type and dimensions can influence the successfulness of EVT. Unfortunately, this information was not available. Strengths of our study are the use of quantified clot characteristics acquired by blinded observers, the size of the data set, and the use of an independent core laboratory.

### Future Directions

We found no evidence that thrombus CT characteristics influence the relationship between first‐line device and outcomes of EVT. However, there was a signal in our data that in fibrin‐rich (low‐density) clots, CA achieves higher rates of FPR than SR, which would be in line with previous studies. Studies using data from the randomized trials should aim to confirm this hypothesis, whereas research focusing on next‐generation devices for those thrombectomy‐resistant clots remains vital to further improve revascularization rates.

## Conclusions

Our study does not provide evidence that the association between first‐line thrombectomy device and EVT outcomes depends on thrombus CT characteristics. Based on our results, there are no arguments for thrombectomy device selection based on thrombus CT characteristics. A possible better performance of CA in low‐density, fibrin‐rich clots needs further study.

## Sources of Funding

This study was funded and carried out by the Erasmus University Medical Center, the Academic Medical Center Amsterdam, and the Maastricht University Medical Center. The study was additionally funded by the European Union's Horizon 2020 Research and Innovation Program grant 777072 (INSIST [In‐Silico Trials for Treatment of Acute Ischemic Stroke]), which played no role in trial design and patient enrollment, nor in data collection, analysis, or writing of the manuscript.

## Disclosures

Erasmus Medical Center received compensation from Stryker, Siemens Healthineers, and GE Healthcare for activities of Dr van der Lugt. Dr Dippel and Dr van der Lugt report grants from the Dutch Heart Foundation, Brain Foundation Netherlands, Health Holland Top Sector Life Sciences & Health, and The Netherlands Organisation for Health Research and Development and unrestricted grants from Stryker, Penumbra Inc., Medtronic, Thrombolytic Science, LLC, and Cerenovus outside the submitted work, all paid to the institution. Dr Marquering is cofounder and shareholder of Nico‐lab, a company that focuses on the use of artificial intelligence for medical image analysis. Dr Majoie is a recipient of grants from the Cardiovascular Research Netherlands (CVON)/Dutch Heart Foundation, Stryker, European Commission, Applied Scientific Institute for Neuromodulation (TWIN) Foundation, and Health Evaluation Netherlands and is a shareholder of Nico‐lab.

## Supporting information



Supporting Information.

## MR CLEAN (Multicenter Randomized Clinical Trial of Endovascular Treatment for Acute Ischemic Stroke in The Netherlands) Registry Investigators–Group Authors

### Executive Committee

Diederik W. J. Dippel,^1^ Aad van der Lugt,^2^ Charles B. L. M. Majoie,^3^ Yvo B. W. E. M. Roos,^4^ Robert J. van Oostenbrugge,^5,41^ Wim H. van Zwam,^6,41^ Jelis Boiten,^14^ Jan Albert Vos^8^

### Study Coordinators

Ivo G. H. Jansen,^3^ Maxim J. H. L. Mulder,^1,2^ Robert‐Jan B. Goldhoorn,^5,6,41^ Kars C. J. Compagne,^2^ Manon Kappelhof,^3^ Josje Brouwer,^4^ Sanne J. den Hartog,^1,2,40^ Wouter H. Hinsenveld^5,6^

### Local Principal Investigators

Diederik W. J. Dippel,^1^ Bob Roozenbeek,^1^ Aad van der Lugt,^2^ Adriaan C. G. M. van Es,^2^ Charles B. L. M. Majoie,^3^ Yvo B. W. E. M. Roos,^4^ Bart J. Emmer,^3^ Jonathan M. Coutinho,^4^ Wouter J. Schonewille,^7^ Jan Albert Vos,^8^ Marieke J. H. Wermer,^9^ Marianne A. A. van Walderveen,^10^ Julie Staals,^5,41^ Robert J. van Oostenbrugge,^5,41^ Wim H. van Zwam,^6,41^ Jeannette Hofmeijer,^11^ Jasper M. Martens,^12^ Geert J. Lycklama À Nijeholt,^13^ Jelis Boiten,^14^ Sebastiaan F. de Bruijn,^15^ Lukas C. van Dijk,^16^ H. Bart van der Worp,^17^ Rob H. Lo,^18^ Ewoud J. van Dijk,^19^ Hieronymus D. Boogaarts,^20^ J. de Vries,^22^ Paul L. M. de Kort,^21^ Julia van Tuijl,^21^ Jo P. Peluso,^26^ Puck Fransen,^22^ Jan S. P. van den Berg,^22^ Boudewijn A. A. M. van Hasselt,^23^ Leo A. M. Aerden,^24^ René J. Dallinga,^25^ Maarten Uyttenboogaart,^28^ Omid Eschgi,^29^ Reinoud P. H. Bokkers,^29^ Tobien H. C. M. L. Schreuder,^30^ Roel J. J. Heijboer,^31^ Koos Keizer,^32^ Lonneke S. F. Yo,^33^ Heleen M. den Hertog,^22^ Tomas Bulut,^35^ Paul J. A. M. Brouwers^34^

### Imaging Assessment Committee

Charles B. L. M. Majoie^3^ (chair), Wim H. van Zwam,^6,41^ Aad van der Lugt,^2^ Geert J. Lycklama à Nijeholt,^13^ Marianne A. A. van Walderveen,^10^ Marieke E. S. Sprengers,^3^ Sjoerd F. M. Jenniskens,^27^ René van den Berg,^3^ Albert J. Yoo,^38^ Ludo F. M. Beenen,^3^ Alida A. Postma,^6,42^ Stefan D. Roosendaal,^3^ Bas F. W. van der Kallen,^13^ Ido R. van den Wijngaard,^13^ Adriaan C. G. M. van Es,^2^ Bart J. Emmer,^3^ Jasper M. Martens,^12^ Lonneke S. F. Yo,^33^ Jan Albert Vos,^8^ Joost Bot,^36^ Pieter‐Jan van Doormaal,^2^ Anton Meijer,^27^ Elyas Ghariq,^13^ Reinoud P. H. Bokkers,^29^ Marc P. van Proosdij,^37^ G. Menno Krietemeijer,^33^ Jo P. Peluso,^26^ Hieronymus D. Boogaarts,^20^ Rob Lo,^18^ Wouter Dinkelaar,^2^ Auke P. A. Appelman,^29^ Bas Hammer,^16^ Sjoert Pegge,^27^ Anouk van der Hoorn,^29^ Saman Vinke^20^

### Writing Committee

Diederik W. J. Dippel^1^ (chair), Aad van der Lugt,^2^ Charles B. L. M. Majoie,^3^ Yvo B. W. E. M. Roos,^4^ Robert J. van Oostenbrugge,^5,41^ Wim H. van Zwam,^6,41^ Geert J. Lycklama à Nijeholt,^13^ Jelis Boiten,^14^ Jan Albert Vos,^8^ Wouter J. Schonewille,^7^ Jeannette Hofmeijer,^11^ Jasper M. Martens,^12^ H. Bart van der Worp,^17^ Rob H. Lo^18^

### Adverse Event Committee

Robert J. van Oostenbrugge^5,41^ (chair), Jeannette Hofmeijer,^11^ H. Zwenneke Flach^23^

### Trial Methodologist

Hester F. Lingsma^40^

### Research Nurses/Local Trial Coordinators

Naziha el Ghannouti,^1^ Martin Sterrenberg,^1^ Wilma Pellikaan,^7^ Rita Sprengers,^4^ Marjan Elfrink,^11^ Michelle Simons,^11^ Marjolein Vossers,^12^ Joke de Meris,^14^ Tamara Vermeulen,^14^ Annet Geerlings,^19^ Gina van Vemde,^22^ Tiny Simons,^30^ Gert Messchendorp,^28^ Nynke Nicolaij,^28^ Hester Bongenaar,^32^ Karin Bodde,^24^ Sandra Kleijn,^34^ Jasmijn Lodico,^34^ Hanneke Droste,^34^ Maureen Wollaert,^5^ Sabrina Verheesen,^5^ D. Jeurrissen,^5^ Erna Bos,^9^ Yvonne Drabbe,^15^ Michelle Sandiman,^15^ Nicoline Aaldering,^11^ Berber Zweedijk,^17^ Jocova Vervoort,^21^ Eva Ponjee,^22^ Sharon Romviel,^19^ Karin Kanselaar,^19^ Denn Barning^10^

### PhD/Medical Students

Esmee Venema,^40^ Vicky Chalos,^1,40^ Ralph R. Geuskens,^3^ Tim van Straaten,^19^ Saliha Ergezen,^1^ Roger R. M. Harmsma,^1^ Daan Muijres,^1^ Anouk de Jong,^1^ Olvert A. Berkhemer,^1,3,6^ Anna M. M. Boers,^3,39^ J. Huguet,^3^ P. F. C. Groot,^3^ Marieke A. Mens,^3^ Katinka R. van Kranendonk,^3^ Kilian M. Treurniet,^3^ Manon L. Tolhuisen,^3,39^ Heitor Alves,^3^ Annick J. Weterings,^3^ Eleonora L. F. Kirkels,^3^ Eva J. H. F. Voogd,^11^ Lieve M. Schupp,^3^ Sabine L. Collette,^28,29^ Adrien E. D. Groot,^4^ Natalie E. LeCouffe,^4^ Praneeta R. Konduri,^39^ Haryadi Prasetya,^39^ Nerea Arrarte‐Terreros,^39^ Lucas A. Ramos^39^

### Thrombus Imaging Committee

Manon Kappelhof,^3,39^ Agnetha A. E. Bruggeman,^3^ Nikki Boodt,^1,2,40^ Praneeta R. Konduri,^39^ Nerea Arrarte‐Terreros,^39^ Jan W. Hoving,^3^ Josje Brouwer^4^

### List of Affiliations

Departments of Neurology,^1^ Radiology,^2^ and Public Health,^40^ Erasmus MC University Medical Center, Departments of Radiology and Nuclear Medicine,^3^ Neurology,^4^ and Biomedical Engineering & Physics,^39^ Amsterdam UMC, University of Amsterdam, Amsterdam, Departments of Neurology^5^ and Radiology & Nuclear Medicine,^6^ Maastricht University Medical Center+, School for Cardiovascular Diseases Maastricht^41^, and School for Mental Health and Neuroscience, Maastricht, The Netherlands^42^, Departments of Neurology^7^ and Radiology,^8^ Sint Antonius Hospital, Nieuwegein, Departments of Neurology^9^ and Radiology,^10^ Leiden University Medical Center, Departments of Neurology^11^ and Radiology,^12^ Rijnstate Hospital, Arnhem, Departments of Radiology^13^ and Neurology,^14^ Haaglanden MC, the Hague, Departments of Neurology^15^ and Radiology,^16^ HAGA Hospital, the Hague, Departments of Neurology^17^ and Radiology,^18^ University Medical Center Utrecht, Departments of Neurology,^19^ Neurosurgery,^20^ and Radiology,^27^ Radboud University Medical Center, Nijmegen, Departments of Neurology^21^ and Radiology,^26^ Elisabeth‐TweeSteden ziekenhuis, Tilburg, Departments of Neurology^22^ and Radiology,^23^ Isala Klinieken, Zwolle, Departments of Neurology^24^ and Radiology,^25^ Reinier de Graaf Gasthuis, Delft, Departments of Neurology^28^ and Radiology,^29^ University Medical Center Groningen, Departments of Neurology^30^ and Radiology,^31^ Atrium Medical Center, Heerlen, Departments of Neurology^32^ and Radiology,^33^ Catharina Hospital, Eindhoven, Departments of Neurology^34^ and Radiology,^35^ Medisch Spectrum Twente, Enschede, Department of Radiology,^36^ Amsterdam UMC, Vrije Universiteit van Amsterdam, Amsterdam, Department of Radiology,^37^ Noordwest Ziekenhuisgroep, Alkmaar, Department of Radiology,^38^ Texas Stroke Institute, Texas, USA
