# Supplementary material for: Value of Thrombus Imaging Characteristics as a Guide for First‐Line Endovascular Thrombectomy Device in Patients With Acute Ischemic Stroke
Source: Stroke Vasc Interv Neurol. 2022 Sep 9;3(1):e000450. doi: 10.1161/SVIN.122.000450 (PMC12778580; doi:10.1161/SVIN.122.000450)

SUPPLEMENTAL MATERIALS

Value of Thrombus Imaging Characteristics as a Guide for First-line Endovascular Thrombectomy Device in Patients with Acute Ischemic Stroke

Nikki Boodt[1,2,3], MD; Agnetha A. E. Bruggeman[4], MD; Manon Kappelhof[4,5], MD, PhD; Sanne J. den Hartog[1,2,3], MD; Nerea Arrarte Terreros[4,5], MSc; Jasper M. Martens[6], MD; Reinoud P. H. Bokkers[7], MD, PhD; Pieter-Jan van Doormaal[1], MD; Charles B. L. M. Majoie[4], MD, PhD; Wim H. van Zwam[8], MD, PhD; Henk A. Marquering[4,5], PhD; Diederik W. J. Dippel[2], MD, PhD; Aad van der Lugt[1], MD, PhD; Hester F. Lingsma[3], MSc, PhD; on behalf of the MR CLEAN Registry Investigators

[1]Erasmus MC, University Medical Center Rotterdam, Department of Radiology and Nuclear Medicine, Rotterdam, the Netherlands

[2]Erasmus MC, University Medical Center Rotterdam, Department of Neurology, Rotterdam, the Netherlands

[3]Erasmus MC, University Medical Center Rotterdam, Department of Public Health, Rotterdam, the Netherlands

[4]Amsterdam UMC, University of Amsterdam, Department of Radiology and Nuclear Medicine, Amsterdam, the Netherlands

[5]Amsterdam UMC, University of Amsterdam, Department of Biomedical Engineering and Physics, Amsterdam, the Netherlands

[6]Rijnstate Hospital, Department of Radiology and Nuclear Medicine, Arnhem, the Netherlands

[7] University of Groningen, University Medical Center Groningen, Medical Imaging Center, Department of Radiology Groningen, the Netherlands

[8]Maastricht University Medical Center, Department of Radiology and Nuclear Medicine, Cardiovascular Research Institute Maastricht (CARIM), Maastricht, the Netherlands

Supplemental Tables

Table I: Overall outcomes for first-line CA versus first-line SR 3
Table II: Outcomes and interaction of HAS and first-line device 4

Supplemental Figures

Figure I: Flowchart of patient inclusion 5
Figure II: *Distribution of thrombus density and thrombus length* 6
Figure III: First-line device per year and per intervention center 7
Figure IV: *Relationship of HAS and first-line device with FPR* 8

Table I. Primary and secondary outcomes for patients treated with first-line contact aspiration versus stent-retriever (reference) thrombectomy.

**Outcome**	**Effect estimate**	**Unadjusted value (95% CI)**	**Adjusted value* (95% CI)**
FPR, n (%)	Odds ratio	1.20 (0.82-1.75)	1.32 (0.88-1.98)
Final eTICI, median (IQR)	Common odds ratio	1.03 (0.76-1.39)	1.05 (0.74-1.49)
Duration of procedure, minutes, median (IQR)	Beta	-14.6 (-20.3 to -8.9)	-14.2 (-20.5 to -8.0)
NIHSS at 24 hours, median (IQR)	Beta	0.96 (-0.50 to 2.41)	0.21 (-0.99 to 1.41)
mRS at 90 days, median (IQR)	Common odds ratio	0.94 (0.70-1.27)	1.12 (0.79-1.59)

Duration of procedure was defined as time from groin puncture to successful reperfusion (eTICI ≥2B) or last contrast bolus when no successful reperfusion was achieved. *Adjusted for age, sex, prestroke mRS, baseline NIHSS, occlusion location, collaterals, intravenous alteplase treatment, time from symptom onset to groin puncture, calendar year and intervention center.

FPR indicates first-pass reperfusion; IQR, interquartile range, eTICI, expanded treatment in cerebral ischemia; duration of procedure, time from groin puncture to eTICI ≥2B or higher or last angiography run in case eTICI ≥2B was not achieved; NIHSS, National Institutes of Health Stroke Scale and mRS, modified Rankin Scale.

Table II. Primary and secondary outcomes for patients treated with contact aspiration versus stent retriever (reference) for patients with and without hyperdense artery sign.

**Outcome**	**Effect estimate**	**HAS- (n=311)**	**HAS+ (n=390)**	***p* for interaction**
FPR	Odds ratio	1.40 (0.74-2.67)	1.18 (0.70-2.01)	0.83
Final eTICI	Common odds ratio	0.88 (0.53-1.45)	1.19 (0.74-1.90)	0.22
Duration of procedure, minutes	Beta	-10.9 (-21.0 to -0.8)	-15.3 (-22.6 to -8.1)	0.57
NIHSS at 24 hours	Beta	1.85 (0.07-3.62)	-0.82 (-2.47 to 0.82)	0.04
mRS at 90 days	Common odds ratio	0.97 (0.57-1.67)	1.17 (0.76-1.81)	0.58

Analyses were adjusted for age, sex, prestroke mRS, baseline NIHSS, occlusion location, collaterals, intravenous alteplase treatment, time from onset to groin, calendar year and intervention center.

FPR indicates first-pass reperfusion; IQR, interquartile range, eTICI, expanded treatment in cerebral ischemia; duration of procedure, time from groin puncture to eTICI ≥2B or higher or last angiography run in case eTICI ≥2B was not achieved; NIHSS, National Institutes of Health Stroke Scale and mRS, modified Rankin Scale.

Figure I. Flowchart of patient inclusion.

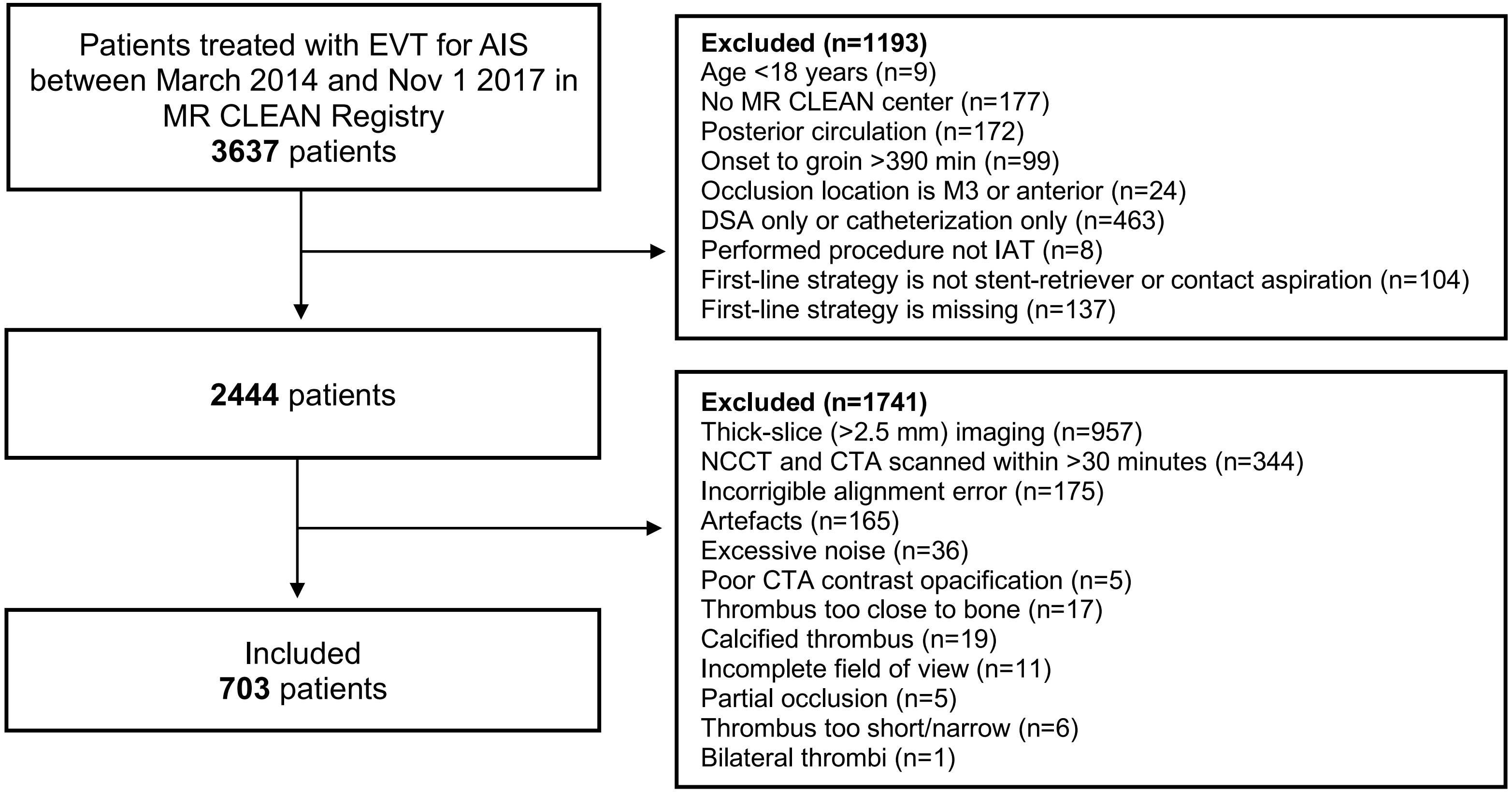

Figure II. Distribution of thrombus density (A) and thrombus length (B).

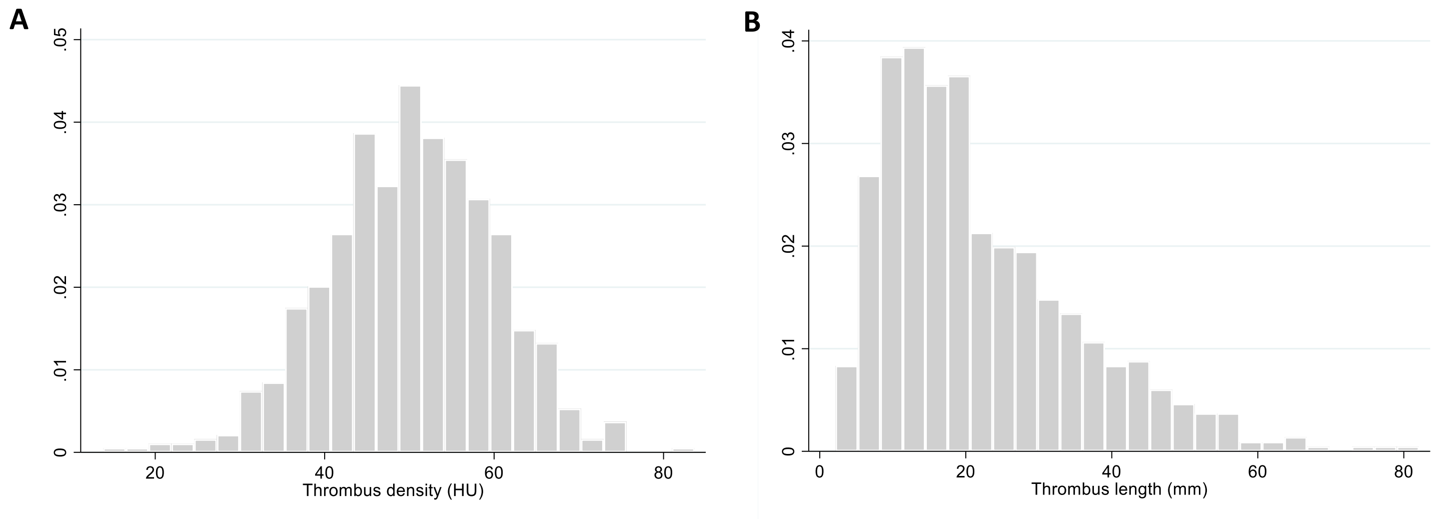

Figure III. First-line thrombectomy modality per inclusion year (A) and per intervention center (B).

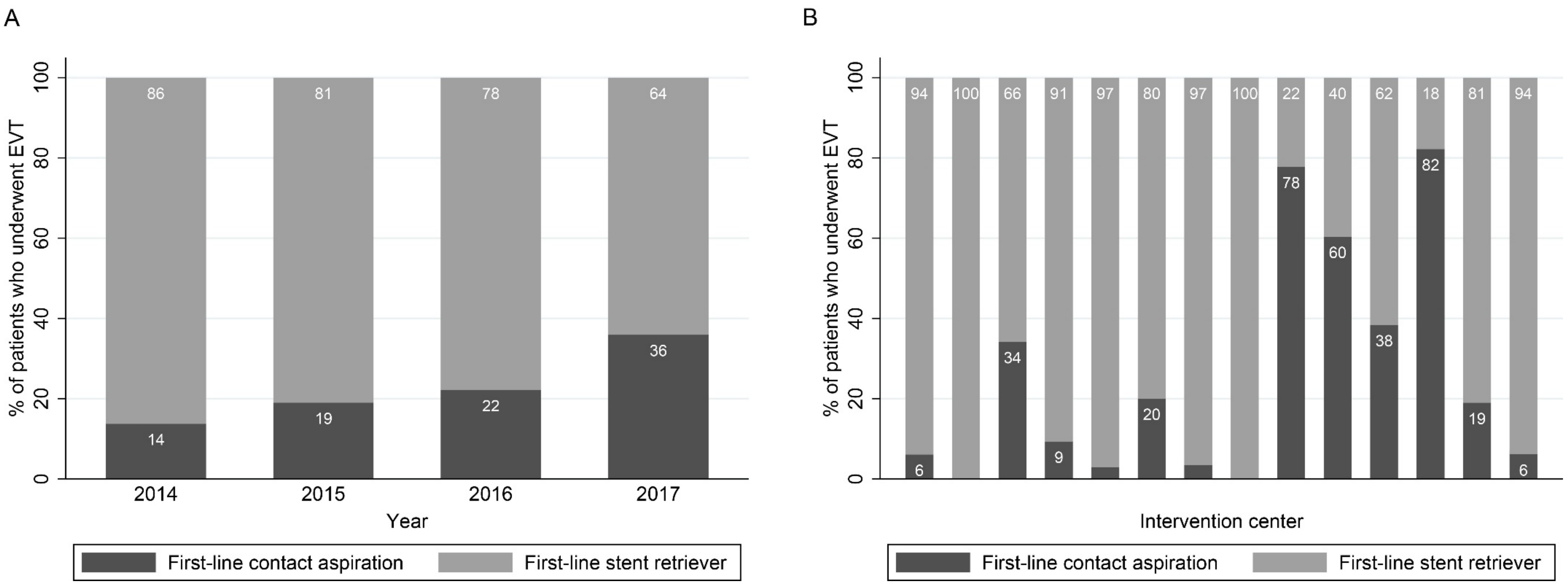

Figure IV. Adjusted margins plot showing the relationship between presence of hyperdense artery sign and first-line thrombectomy modality with the primary outcome, first-pass reperfusion (FPR).

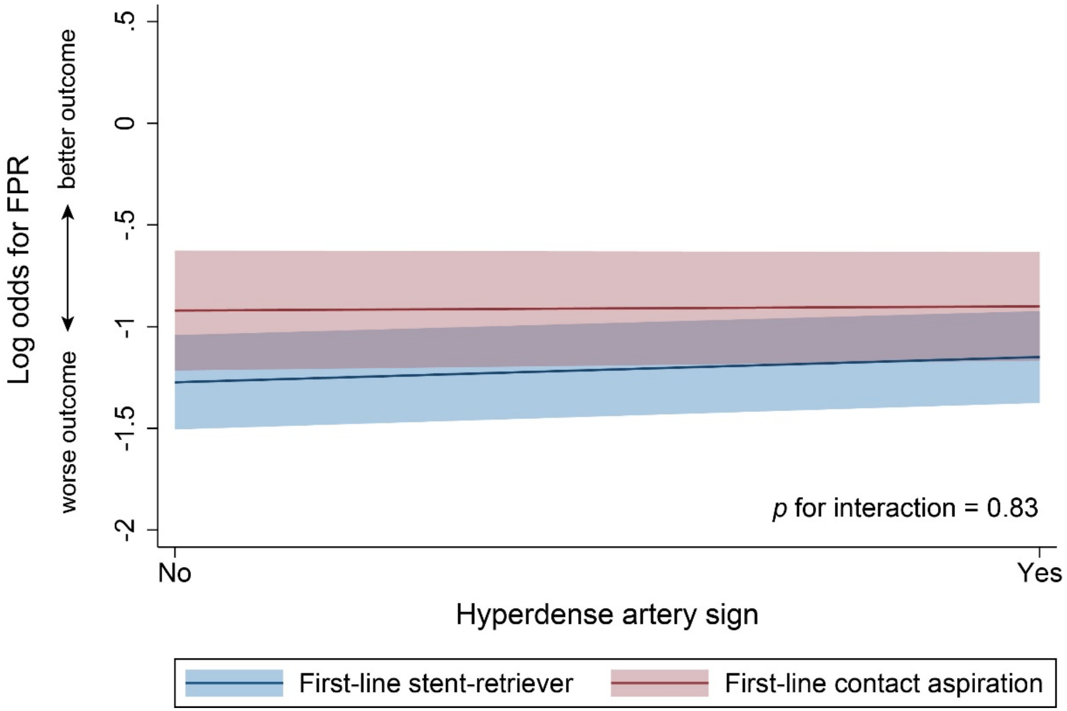

Supplement: Supplementary file 1 — Supporting Information. [file SVI2-3-e000450-s001.pdf]
